# Supplementary material for: The Expression of miRNAs Involved in Long-Term Memory Formation in the CNS of the Mollusk Helix lucorum
Source: Int J Mol Sci. 2022 Dec 24;24(1):301. doi: 10.3390/ijms24010301 (PMC9820140; doi:10.3390/ijms24010301)
Supplement: Supplementary file 1 [file ijms-24-00301-s001.zip › ijms-2014974-supplementary/Supplementary File S3. Alignement.pdf]

Alignments of human and mollusks mature microRNAs belonging to the families MIR-10, -33, -76, -92, -133, -137, -193, -277, -1985.
Species designations: Hlu – Helix lucorum, Hsa – Homo sapiens, Lgi – Lottia gigantea, Cgi – Crassostrea gigas, Aca – Aplysia californica.

```
Hlu-Mir-10-S5_5p       AACCCGUAGAACCGAACUUGU-
Hlu-Mir-10-S6_5p       AACCCGUAGA-CCGAACUUGU-
Hsa-miR-10-P2d-5p      AACCCGUAGAUCCGAACUUGUG
Lgi-Mir-10-P2_5p       AACCCGUAGAUCCGAACUUGUG
Cgi-Mir-10-P2_5p       AACCCGUAGAUCCGAACUUGUG
Aca-Mir-10-P2d_5p      AACCCGUAGAUCCGAACUUGUG
                       ********** **********

Hlu-Mir-10-S9_5p       UCCCUGAGACCAUAAUUUGAGC
Hlu-Mir-10-S10_5p      ACCCUGAGACCAUAAUUUGAUC
Hlu-Mir-10-S12_5p      UCCCUGAGACCAUAAUU-GAGC
Hlu-Mir-10-S18_5p      UCCCUGAGACCAAAAUUUGUGC
Hlu-Mir-10-S19_5p      UCCCUGAGACUAUAAUUUGUGC
Hsa-Mir-10-P3d_5p      UCCCUGAGACCCUAACUUGUGA
Lgi-Mir-10-P3_5p       UCCCUGAGACCAUAAUUUUGAU
Cgi-Mir-10-P3_5p       UCCCUGAGACCAUAACUUGUGA
Aca-Mir-10-P3d_5p      UCCCUGAGACCCUAACUUGUGA
                        *********   ** * *

Hlu-Mir-33_5p          GUGCAUUGUAGUUGCAUUGCC
Hsa-Mir-33-P3_5p       GUGCAUUGUAGUUGCAUUGCA
Lgi-Mir-33_5p          GUGCAUUGUAGUUGCAUUGCA
Cgi-Mir-33_5p          GUGCAUUGUAGUUGCAUUGCA
Aca-Mir-33-P3_5p       GUGCAUUGUAGUUGCAUUGCA
                       ********************

Hlu-Mir-76-S2_3p       UUCGUUGUCGUCGAAACAUGACU
Hlu-Mir-76-S3_3p       UUCGUUGUAUUCGAAACCUGCCU
Lgi-Mir-76_3p          UUCGUUGUCGACGAAACCUGCCU
Cgi-Mir-76_3p          UUCGUUGUCGUCGAAACCUGCCU
                       ********   ****** ** **

Hlu-Mir-92-S1_3p       AAUUGCACUCGUCCCGGCCUGC
Hlu-Mir-92-S2_3p       CAUUGCACUUAUCCCGGCCUCA
Hlu-Mir-92-S3_3p       UAUUGCACUCGUCCCGGCCUUC
Hlu-Mir-92-S4_3p       CAUUGCACUUAUCCCGGCUUGA
Hsa-Mir-92-P1d_3p      UAUUGCACUCGUCCCGGCCUCC
Lgi-Mir-92-o30_3p      AAUUGCACUUGUCCCGGCCUGC
Cgi-Mir-92-o33_3p      UAUUGCACUCGUCCCGGCCGAU
Aca-Mir-92-P1c_3p      UAUUGCACUUGUCCCGGCCUGU
                        ********  *******
```

```
Hlu-Mir-133_3p            UUGGUCCCCUUCAAUCAGUUGU
Hsa-Mir-133-P1-v2_3p      UUGGUCCCCUUCAACCAGCUGU
Lgi-Mir-133_3p            UUGGUCCCCUUCAACCAGCUGU
Cgi-Mir-133_3p            UUGGUCCCCUUCAACCAGCUGU
Aca-Mir-133-P1-v2_3p      UUGGUCCCCUUCAACCAGCUGU
                          ************** *** ***

Hlu-Mir-137_3p            UAUUGCUUGAGAAUACACGUAA-
Hsa-Mir-137-P1-v2_3p      UAUUGCUUAAGAAUACGCGUAGU
Lgi-Mir-137_3p            UAUUGCUUGAGAAUACACGUAA-
Cgi-Mir-137_3p           UUAUUGCUUGAGAAUACACGU
Aca-Mir-137-P3-v2_3p      UAUUGCUUGAGAAUACGCGUAGU
                          ******** ******* ****

Hlu-Mir-193_3p            UACUGGCCUUCAAAAUCCCAAA
Hsa-Mir-193-P1a_3p        AACUGGCCCUCAAAGUCCCGCU
Lgi-Mir-193-P1_3p         UACUGGCCUGCAAAAUCCCAACU
Cgi-Mir-193-P1_3p         UACUGGCCUGCAAAAUCCCAAA
Aca-Mir-193-P1b_3p*       AACUGGCCUACAAAGUCCCAGU
                           *******  **** ****

Hlu-Mir-277_3p            UAAAUGCAUUAUCUGGUAUCUG
Lgi-Mir-277_3p            UAAAUGCAUUUUCUGGUAUCUGA
Cgi-Mir-277_3p            UAAAUGCAUAAUCUGGUAUGUGA
                          *********  ******** **

Hlu-Mir-1985-S1_5p        UGCCAUUUUUAUCAGUCACUGU
Lgi-Mir-1985_5p           UGCCAUUUUUAUCAGUCACUGUG
Cgi-Mir-1985_5p           UGCCAUUUUUAUCAGUCACUGUG
                          **********************
```
